# O-GlcNAc Transferase Inhibition Differentially Affects Breast Cancer Subtypes

**DOI:** 10.1038/s41598-019-42153-6

**Published:** 2019-04-05

**Authors:** Anna Barkovskaya, Kotryna Seip, Bylgja Hilmarsdottir, Gunhild M. Maelandsmo, Siver A. Moestue, Harri M. Itkonen

**Affiliations:** 1 Department of Tumor Biology, The Norwegian Radium Hospital, Institute for Cancer Research, Oslo University Hospital, Oslo, Norway; 2 Department of Circulation and Medical Imaging, NTNU - Norwegian University of Science and Technology, Trondheim, Norway; 3 Institute of Clinical Medicine, Faculty of Medicine, University of Oslo, Oslo, Norway; 4 Faculty of Health Sciences, Institute of Medical Biology, The Arctic University of Norway – University of Tromsø, Tromsø, Norway; 5 Department of Health Sciences, Nord University, Bodø, Norway; 6 Department of Microbiology, Blavatnik Institute, Harvard Medical School, Boston, MA USA

## Abstract

Post-translational modification of intracellular proteins with a single N-acetylglucosamine sugar (O-GlcNAcylation) regulates signaling, proliferation, metabolism and protein stability. In breast cancer, expression of the enzyme that catalyzes O-GlcNAcylation – O-GlcNAc-transferase (OGT), and the extent of protein O-GlcNAcylation, are upregulated in tumor tissue, and correlate with cancer progression. Here we compare the significance of O-GlcNAcylation in a panel of breast cancer cells of different phenotypes. We find a greater dependency on OGT among triple-negative breast cancer (TNBC) cell lines, which respond to OGT inhibition by undergoing cell cycle arrest and apoptosis. Searching for the cause of this response, we evaluate the changes in the proteome that occur after OGT inhibition or knock-down, employing a reverse-phase protein array (RPPA). We identify transcriptional repressor - hairy and enhancer of split-1 (HES1) - as a mediator of the OGT inhibition response in the TNBC cells. Inhibition of OGT as well as the loss of HES1 results in potent cytotoxicity and apoptosis. The study raises a possibility of using OGT inhibition to potentiate DNA damage in the TNBC cells.

## Introduction

Breast cancer is the most prevalent cancer type and the second leading cancer-related cause of death in women^1^. Course of treatment and prognosis depend on the histopathological evaluation of the hormone receptor status - estrogen receptor-α (ER), progesterone receptor (PR) and over-expression of human epidermal growth factor receptor 2 (HER2)^2,3^. 10–20% of breast cancers lack expression of the hormone receptors and do not over-express HER2. This sub-group is referred to as triple-negative breast cancers (TNBC)^4^. These tumors frequently have a basal-like phenotype, mutations in BRCA1 and tend to be more aggressive and invasive^5,6^. Patients with TNBC tumors do not stand to benefit from hormone therapy or HER2 inhibitors^7^, leaving them with limited therapeutic options and poor overall survival^6,8^.

O-GlcNAcylation is a post-translational protein modification. A sole known enzyme, O-GlcNAc transferase (OGT), catalyzes the transfer of β-N-acetylglucosamine (O-GlcNAc) from UDP-GlcNAc onto serine and threonine residues of intracellular proteins^9^. Protein O-GlcNAcylation is reversible; removal of O-GlcNAc is catalyzed by the enzyme termed N-Acetyl-Beta-D-Glucosaminidase (OGA)^10–12^. Previous studies have implicated protein O-GlcNAcylation in promotion of the cancer hallmarks by sustaining growth and invasion^13^, regulating DNA damage- and stress-responses^14,15^ and controlling cell cycle progression^16–18^. O-GlcNAcylation is increased in most malignant tumors, including breast cancer, where it positively correlates with tumor progression^18,19^. It has been shown that both ER^20,21^ and PR^22^ are O-GlcNAcylated. An increase of the total O-GlcNAc levels, achieved through inhibition of OGA, can protect breast cancer cells from ER inhibitors^23^. However, it is not known if certain breast cancer subtypes are more dependent on protein O-GlcNAcylation or whether inhibition of OGT could be a useful therapeutic opportunity for some of the patients.

Here, we set out to compare the impact of OGT inhibition on proliferation and survival of breast cancer cells of different subtypes. In a panel of two receptor-positive and five TNBC cell lines, inhibition of OGT, either with a small-molecule inhibitor or through an siRNA-mediated knock-down, led to a more prominent cell death and growth inhibition in the TNBC cells. To understand the nature of the higher sensitivity of the TNBC cells to OGT inhibition, we performed reverse phase protein array (RPPA) profiling. We identified a transcriptional repressor − hairy and enhancer of split-1 (HES1) as a protein selectively down-regulated in TNBC, but not in the receptor-positive cells in response to OGT inhibition. Knock-down of HES1 phenocopied cytotoxicity observed after OGT inhibition in TNBC cells. HES1 appears to have a specific role in TNBC cells, having a strong association with poor survival in this patient group.

## Materials and Methods

### Cell culture

Cell lines were purchased from ATCC (Rockville, MD), maintained in humidified incubators at 37 C° with 5% CO_2_ atmosphere and routinely tested for mycoplasma infections (PCR mycoplasma detection kit, Minerva Biolabs, Germany). Cells were cultured in the following media: MDA-MB-231 in RPMI-1640 supplemented with 5% fetal bovine serum (FBS) and 2 mM L-Alanyl L-Glutamine (g-max) (all purchased from Sigma Aldrich, St.Louis, MO); BT549 in RPMI-1640 with 10% FBS, g-max and 1 µg/ml human insulin (Sigma Aldrich); MCF7 in DMEM (Sigma Aldrich) with 10% FBS; T74D, MDA-MB-468, HCC38 and HCC70 in RPMI with 10% FBS and g-max. Cell line ID testing was performed by Genetica Labcorp (Burlington, NC).

### Viability and cell proliferation assays

To evaluate the viability after treatment with inhibitors, cells were plated into 96-well plates using 5 × 10^4^ MDA-MB-231, MDA-MB-468 and BT549, 3 × 10^4^ HCC38, HCC70, MCF7 and T47D cells per well in a volume of 100 µl. The following day, the 2x concentrated inhibitors were introduced in 100 µl of fresh respective growth media. Following 72 hours of incubation, the viability was measured using the CellTiter 96® AQ_ueous_ One Solution (Promega, Madison, WI) Cell Proliferation Assay (MTS) according to the supplier’s protocol. To assess cell proliferation, the plates were kept in the IncuCyte® instrument (Essen BioScience, Hertfordshire, UK) which took images of each well every 3 hours and generated estimates of cell density expressed as percent of the total well area occupied by the cells.

### Transient knockdowns

Reverse siRNA knockdowns were performed using two OGT siRNAs: s16094 and s16095; and two HES1 siRNAs: s6920 and s6921 (Thermo Fisher Scientific, Rockford, IL). 10 nM siRNA was mixed with 2 µl of Lipofectamine 3000® reagent (Invitrogen, Carlsbad, CA) in 360 µl Opti-MEM® reduced serum medium (Thermo Fisher Scientific) per well. The mixture was incubated for 20–30 minutes in room temperature and placed into 6-well culture plates. The cells were added to the plates in 2 ml of respective cell culture media, using 1,5 × 10^5^ MDA-MB-231 and MDA-MB-468; 1,8 × 10^5^ BT-549; 1,2 × 10^5^ MCF7 or T47D cells per well.

### Western blotting, immunoprecipitation and RPPA

For western blotting, cells were collected on ice using a scraper, washed in PBS and lyzed in RIPA buffer (50 mM Tris-HCl, pH7.4; 1% NP-40; 0.5% sodium-deoxycholate; 0.1% SDS; 150 mM NaCl) supplemented with phosphatase and protease inhibitors (Roche Applied Science, Mannheim, Germany) and 50 µM OGA inhibitor (PUGNAc, Sigma Aldrich). Following ultra-sonification, protein concentration of the samples was measured using the BCA protein assay kit (Pierce™, Thermo Scientific). 15–20 µg of protein was loaded per each gel lane. Membranes were visualized using the super signal West Dura kit (Thermo Scientific) in the Syngene G:Box instrument (Syngene, Cambridge, UK) with GeneSnap software. Densitometry was performed using ImageJ.

Reverse phase protein array (RPPA) was performed at the core facility of functional proteomics in MD Anderson Cancer Center, University of Texas, Houston TX. Protein lysate samples were prepared for analysis in accordance with the core facility’s instructions *(available at* https://www.mdanderson.org/research/research-resources/core-facilities/functional-proteomics-rppa-core.html). Protein concentration was adjusted to 1.5 µg/ml, and 35–50 µl of each sample was submitted for analysis.

### Gene expression analysis

For gene expression analysis, cells were collected on ice after 24 hours of treatment. RNA was isolated using illustra RNA spin mini kit (GE healthcare, Chicago, IL), following the supplier’s instructions. RNA concentration was measured using NanoDrop™ (Thermo Fisher Scientific). cDNA was derived using the qScript™ cDNA Synthesis Kit (Quanta Biosciences, Gaithersburg, MD), cDNA stock concentration was adjusted to 7.14 ng/µl. Each qPCR reaction contained 2.5 µl of stock cDNA, 2.5 ul of 1 µM forward and reverse primer mix and 5 µl of Fast SYBR™ Green master mix (Thermo Fischer Scientific). The qPCR reaction was performed using a Bio-Rad CFX Connect™ Real Time PCR machine (Bio-Rad, Hercules, CA).

### Cell cycle, TUNEL assay and Annexin V assay

Cells were collected on ice, washed with cold PBS and immediately fixed in ice-cold 100% methanol. Samples were stored at −20 °C until further use. TUNEL assay was performed using Terminal Transferase and Biotin-16-dUTP (both from Roche Diagnostics GmbH, Mannheim, Germany), following the supplied protocol supplemented with 10 mM DTT. Following the apoptosis staining, cells were incubated for 30 minutes at 37 C° with the 1.5 µg/ml Hoechst 33258 in PBS solution. This allowed for simultaneous analysis of the cell cycle. Samples were analyzed on the LSR II flow cytometer (BD Bioscience, San Jose, CA).

Annexin V flow-cytometry based assay was also used to measure the proportion of cells undergoing apoptosis (Trevigen®, Gaithersburg, MD). In the assay, the double-negative cells represent normal live cells with an intact membrane. Propidium iodide (PI) - positive cells have an impaired membrane and represent the cells going through either necrosis or late-stage apoptosis. FITC-conjugated Annexin V detects phosphatidylserine - a marker of apoptosis exposed on the outer surface of the cells.

### Primers, antibodies and chemical reagents

The following primer sequences were used: OGT forward: CAGCATCCCAGCTCACTT, reverse: CAGCTTCACAGCTATGTCTTC; HES1 forward: ACGTGCGAGGGCGTTAATAC, reverse: GGGGTAGGTCATGGCATTGA; OGA forward: CGAGTGAACATTCCCATCACT, reverse: CCCAAAGGAGCACAGATGTT; PTEN forward: CGAACTGGTGTAATGATATGT, reverse: CATGAACTTGTCTTCCCGT.

The following antibodies from Cell Signaling Technology (Danvers, MA) were used: anti-OGT (#5368), anti-HES1 (#11988), anti-phospho-Ser235/236-S6 (#4858), anti-γ-H2AX (#9718), anti-PARP (#9542), and anti-Histone 3 (#4499). Mouse-derived anti-O-Linked N-Acetylglucosamine (RL2) (ab2739) was acquired from Abcam (Cambridge, UK). Mouse anti-α-tubulin (#CP06) was from Merck Millipore (Burlington, MA).

The following chemical compounds were used: OSMI1 compound was kindly provided by Professor Suzanne Walker (Harvard Medical School). Additional OSMI1 compound was purchased from Sigma Aldrich (SML1621). Efficacy of the two OSMI1 stocks was determined to be similar (data not shown); O-GlcNAcase inhibitor PUGNAc (A7229), MG132 proteasome inhibitor (M7449), mTORC1 inhibitor Everolimus (Ev) (SML2282) and PR inhibitor mifepristone (MF) (M8046), were purchased from Sigma Aldrich. γ-secretase inhibitor (GSI) RO4929097 was purchased from Selleckchem (S1575).

### Generation of survival plots

Survival plots were generated using the online KM-plotter resource^24^. ER and PR expression were specified to separate the patient groups of interest. Systemically untreated patients were excluded from analysis.

## Results

### Hormone receptor-negative breast cancer cells are sensitive to OGT inhibition

**Figure 1:**
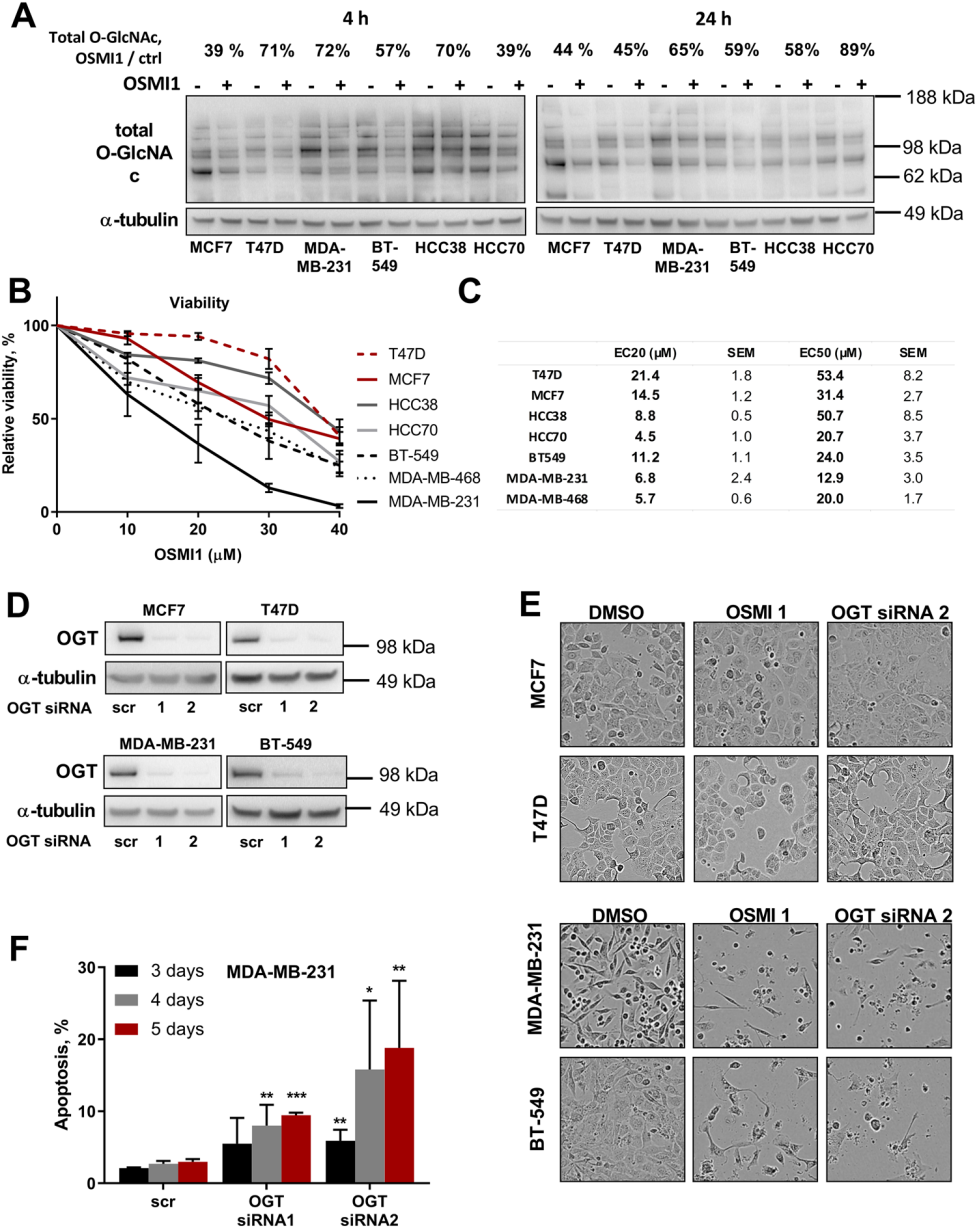
OGT inhibition is potently cytotoxic in TNBC cell lines. (**A**) Relative decrease in total protein O-GlcNAcylation, after 4 and 24 hours of treatment with 40 µM OSMI1 in six BC cell lines. Percentage above indicates median decrease compared to the respective DMSO-treated controls, at least 2 independent experiments. (**B**) Relative viability in BC cell lines after 72 hours of treatment with OSMI1. MTS assay. At least 3 biological replicates for each cell line. Error bars - SEM. TNBC lines marked in black and grey; triple-positive cell lines in red. (**C**) EC50 and EC20 concentrations of OSMI1, mean from at least three independent experiments for each cell line. (**D**) OGT transient knock-down in four BC cell lines, representative out of at least three experiments. (**E**) BC cells pictured after 72 hours of treatment with 20 µM OSMI1, or 5 days of transient OGT knockdown. (**F**) Apoptotic population in MDA-MB-231 following 3, 4 and 5 days of transient OGT knock-down, TUNEL assay. Error bars - standard deviation, n = at least 2. *p < 0.05; **p < 0.005; ***p < 0.001 unpaired t-test, comparing knock-down samples to the respective scr RNA control samples.

Importance of OGT in breast cancer has been previously reported, however the diversity and molecular heterogeneity of breast cancer has never been taken into account. Here we compare the impact of OGT inhibition on a panel of breast cancer cells with a different hormone receptor status: ER- and PR-positive MCF7 and T47D; and receptor-negative MDA-MB-231, BT-549, MDA-MB-468, HCC38 and HCC70. In line with the previous reports, the OGT inhibitor, OSMI1^25^, caused a decrease in total protein O-GlcNAcylation (Fig. 1A). Treatment caused a consistent 30–60% decrease in total O-GlcNAcylation in all of the tested cell lines, and the response did not depend on the hormone receptor status.

After confirming that OSMI1 is able to inhibit OGT activity, we moved on to assess the effect of OGT inhibition on cell viability. OSMI1 strongly decreased the viability in all TNBC cell lines, with an average EC_20_ value of ~7 µM (Fig. 1B,C). The strongest effect was observed in the MDA-MB-231 cell line, where treatment with 40 µM OSMI1 led to a complete loss of viability. Notably, the effect on the receptor-positive cell lines MCF7 and T47D, was less prominent, with the average EC_20_ value more than two times higher than in the TNBC cells, ~18 µM. Both the OGT knock-down and the treatment with OSMI1 altered cell morphology (Fig. 1D,E), indicating possible cell death in MDA-MB-231 and BT-549 cells, but not in the receptor-positive MCF7 and T47D cells. In order to test if the cell death was triggered in response to OGT inhibition, we measured apoptosis in the MDA-MB-231 cells using TUNEL assay following a transient OGT knock-down. Indeed, we found a time-dependent induction of apoptosis (Fig. 1F). TUNEL assay detects DNA strand breaks that occur during apoptosis, however the DNA strand breaks can in some cases be repaired^26^. Therefore, we detected total length and cleaved PARP in order to corroborate the induction of apoptosis^27^. Both of the OGT-targeting siRNAs induced PARP cleavage, thereby confirming apoptosis in response to the OGT knockdown (Supplementary Fig. S1A).

Cell toxicity caused by the OGT inhibition was more prominent in the TNBC cells, while the effect on the receptor-positive MCF7 and T47D cells was much less pronounced. These data suggest that the hormone receptor status appears to be a strong determinant of sensitivity to OGT inhibition.

### Progesterone receptor inhibitor sensitizes hormone receptor-positive cells to OSMI1

**Figure 2:**
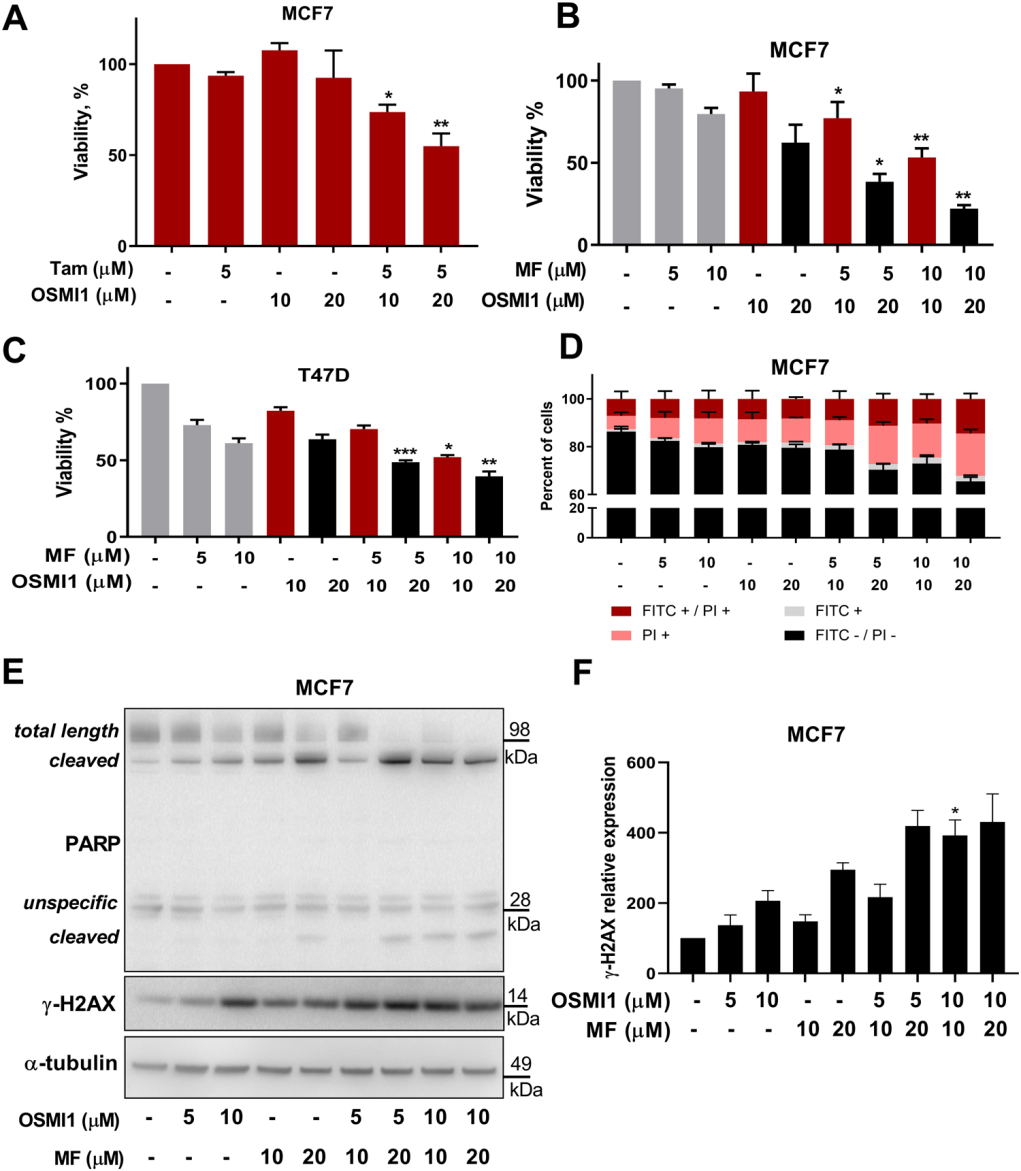
Progesterone receptor inhibitor sensitizes receptor-positive BC cells to OGT inhibition. (**A**) Relative viability in MCF7 after 72 hours of treatment with Tamoxifen (Tam), OSMI1, or a combination. MTS assay. Error bars - SEM, n = 3. *p < 0.05; **p < 0.005, unpaired t-test, comparing viability after the combination treatment to the viability after treatment with respective dose of tamoxifen. (**B**,**C**) Viability in MCF7 and T47D cells following 72 hours of treatment with Mifepristone (MF), OSMI1 or a combination, MTS assay. Error bars - SEM, n = 3. *p < 0.05; **p < 0.005; ***p < 0.001 unpaired t-test, comparing viability after the combination-treatment to the viability after treatment with respective dose of MF. (**D**) Annexin V apoptosis assay in MCF7 following 72 hours of treatment with MF, OSMI1 or a combination. FITC-/PI- population represents live cells; FITC+ cells are in early apoptosis; FITC+/PI+ cells are in late apoptosis; PI+ cells are necrotic. Error bars - standard deviation, n = 2. (**E**) Total length and cleaved PARP and γ-H2AX in MCF7 after 72 hours of treatment with MF, OSMI1, or a combination. Representative of two separate experiments. (**F**) Relative γ-H2AX protein expression in MCF7 following 72 hours of treatment with MF, OSMI1 or a combination. n = 3, error bars - SEM; unpaired t-test, comparing expression after the combination treatments to the expression after respective concentration of MF and OSMI1; *p < 0.05.

Focusing on the difference in the hormone receptor status between the cell lines, we set out to test if the hormone receptor inhibitors may be used to sensitize receptor-positive cells to OGT inhibition. First, we evaluated the effect of an ER inhibitor, tamoxifen, alone or in combination with OSMI1 on the viability of MCF7 cells. Tamoxifen modestly sensitized cells to OSMI1 (Fig. 2A), however the overall loss of cell viability was mild.

Next, we examined the combined effect of PR inhibitor, MF^28^ with OSMI1. A combination of MF with OSMI1 led to an 80% decrease of cell viability (Fig. 2B). We confirmed that MF similarly sensitized another hormone receptor-positive cell line, T47D to OSMI1 (Fig. 2C). As expected, we did not observe an enhanced effect in the TNBC cell lines (Supplementary Fig. S1B).

OGT inhibition resulted in activation of cell death in MDA-MB-231 cells (Fig. 1F, Supplementary Fig. S1A). This prompted us to test if MF is able to enhance cell death induction by OSMI1 in the receptor-positive cells. Using Annexin V flow-cytometry assay, we found that the combination of OSMI1 with MF induced prominent cell death (Fig. 2D), as well as severe morphological changes indicative of cell death in MCF7 cells (Supplementary Fig. S1C). We noted an accumulation of propidium iodide - positive cells, which represents either cells going through late-stage apoptosis or necrosis^29^. We evaluated whether the cell death occurs through apoptosis or necrosis by using PARP cleavage-signature as a marker for these^27,30^. OSMI1-MF combination induced prominent accumulation of PARP fragments of 90 and 25 kDa (Fig. 2E), which are indicative of apoptosis. However, we did not observe accumulation of PARP cleavage fragments typical for necrosis. Based on these data, inhibition of PR sensitizes receptor-positive BC cells to OSMI1.

Next, we sought to understand the mechanism of this combined effect. PR has been shown to protect breast cancer cells from the radiation-induced cell death^31^, while OGT is known to suppress expansion of the DNA damage signaling^15^. These studies led us to hypothesize that the combined inhibition of OGT and PR results in DNA damage. To test this, we evaluated the induction of the canonical DNA damage marker γ-H2AX^32^. Indeed, OSMI1 significantly potentiated the DNA-damage induced by MF alone (Fig. 2E,F).

### OGT inhibition causes cell cycle arrest in breast cancer cells

**Figure 3:**
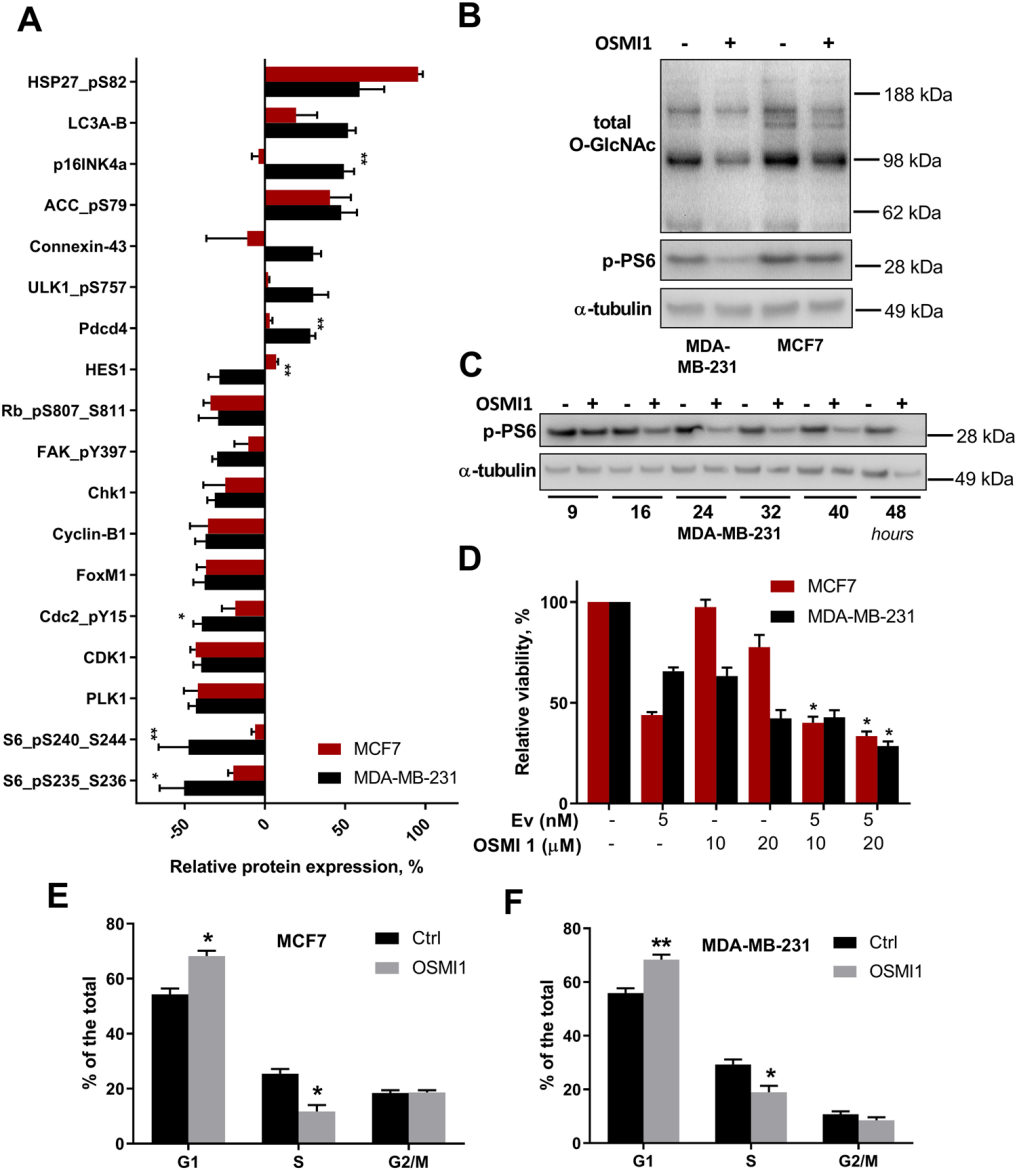
OGT inhibition differentially affects proteome and phospho-proteome in MCF7 and MDA-MB-231. (**A**) Proteins whose expression changed by 28% or more compared to the control samples in MDA-MB-231 following 24 hours of treatment with 20 µM OSMI1, were selected. Corresponding proteins in MCF7 are shown in the same figure. RPPA array, n = 3, error bars - SEM. *p < 0.05; **p < 0.01, unpaired t-test. Significance indicates comparison of the relative protein expression changes in MDA-MB-231 and MCF7. (**B**) Total O-GlcNAcylation and phospho-PS6 (S235-S326) in MDA-MB-231 and MCF7 following treatment with 20 µM OSMI1 for 24 hours. (**C**) Phospho-PS6 (S235-S236) in MDA-MB-231 after treatment with 20 µM OSMI1 for 9, 16, 24, 32, 40 and 48 hours. (**D**) Viability in MCF7 and MDA-MB-231 following 72 hours of treatment with Everolimus (Ev), OSMI1 and the combination. MTS assay, error bars - SEM, n = 3. *p < 0.05, unpaired t-test. (**E**,**F**) Cell cycle distribution in MCF7 (**E**) and MDA-MB-231 (**F**), after 24 hours of treatment with 20 µM OSMI1. n = 3, error bars - SEM. *p < 0.05; **p < 0.005, unpaired t-test.

To better understand the consequences of OGT inhibition in breast cancer cells, we compared the immediate effects on the proteome of the TNBC cells (MDA-MB-231) and the receptor-positive cells (MCF7). Both cell lines were treated with 20 µM OSMI1 for 24 hours, or OGT siRNAs for 72 hours. Following the treatment, proteins were extracted and analyzed with an RPPA assay. To understand the high sensitivity of MDA-MB-231 cells to OGT inhibition, we selected the most up- and down-regulated proteins in this cell line, evaluating the relative changes in protein expression after treatment with OSMI1 (Fig. 3A) and the OGT knock-down (Supplementary Fig. S2A). The most down-regulated protein was the phosphorylated ribosomal protein S6 (p-PS6). The functional role of PS6 phosphorylation remains a matter of debate, but it has been implicated in the regulation of cell size and glucose homeostasis^33^. We confirmed that as the total O-GlcNAcylation decreases after treatment with OSMI1, the p-PS6 is strongly down-regulated in MDA-MB-231, but not in MCF7 (Fig. 3B). This effect is present as early as 16 hours following the treatment and results in a complete loss of p-PS6 after 48 hours (Fig. 3C).

PS6 is phosphorylated in response to activation of the mammalian target of rapamycin complex 1 (mTORC1)^34^. To test whether PS6 phosphorylation mediates the cytotoxic response to OSMI1 treatment in the TNBC cells, we attempted to inhibit mTORC1 to sensitize the MCF7 cells to OGT inhibition. We treated both cell lines with OSMI1 alone and in combination with an mTORC1 inhibitor, Everolimus (Ev). mTORC1 inhibition strongly decreased the viability of the MCF7 cells, however, no combined effect of the dual treatment with OSMI1 was present (Fig. 3D).

In order to identify the pathways that control the increased sensitivity of MDA-MB-231 cells to OGT inhibition, we submitted the list of proteins most differentially regulated by treatment with OSMI1, for the STRING pathway enrichment analysis^35^. Regulation of the mitotic cell cycle was identified as the most affected pathway (Supplementary Fig. S2B,D). We evaluated changes in the cell cycle distribution following OGT inhibition with OSMI1 using flow-cytometry. There was a significant increase in the number of cells in G1/G0 phase, along with a decrease in the number of cells in the S phase. Notably, OSMI1 equally impacted cell cycle phase distribution in both the MDA-MB-231 and the MCF7 cell lines (Fig. 3E,F).

In conclusion, we identified changes in the cell cycle as a major response to OGT inhibition. However, treatment with OSMI1 caused cell cycle arrest in both hormone receptor-negative and -positive cells. This response can therefore not explain the higher sensitivity of TNBC cells to OGT inhibition, indicating that other factors contribute to the differential response.

### Regulation of HES1 in triple-negative breast cancer cells

**Figure 4:**
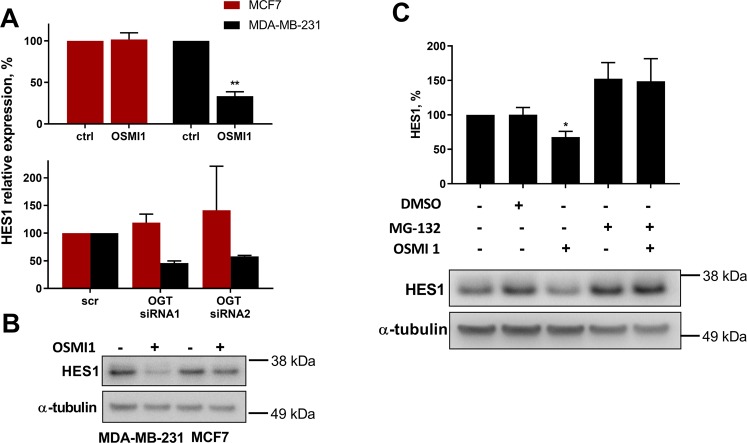
HES1 is selectively regulated by OGT in TNBC cells. (**A**) Relative protein HES1 expression following 24 hours of treatment with 20 µM OSMI1 (upper panel, error bars: SEM, n = 4; **p < 0.005, unpaired t-test) or 72 hours after a transient OGT knock-down (lower panel, error bars: St. dev, n = 2). (**B**) HES1 in MDA-MB-231 and MCF7 after 24 hours of treatment with 20 µM OSMI1. (**C**) HES1 protein expression in MDA-MB-231 following 2 hours of treatment with OSMI1, proteasome inhibitor MG-132, DMSO or combinations. Error bars - SEM, n = 3; *p < 0.05, unpaired t-test.

Searching for potential mediators of high sensitivity of the TNBC cells to OGT inhibition, we identified transcriptional repressor HES1, as a protein that was strongly decreased in TNBC, but not in the receptor-positive cells (Fig. 3A). In the RPPA data, HES1 was down-regulated by 30% in MDA-MB-231 following both the treatment with OSMI1 and the OGT knock-down. In MCF7, however, HES1 was mildly increased after treatment with OSMI1 and slightly down-regulated after the OGT knock-down (Fig. 3A, Supplementary Fig. S2A). We confirmed that both OSMI1 and OGT knock-down caused a greater than 50% decrease in HES1 protein expression in the TNBC cells, while HES1 expression in the receptor-positive cells remained unchanged, or modestly elevated (Fig. 4A,B). In addition, HES1 was prominently down-regulated in all tested TNBC cell lines following 24 hours of treatment with OSMI1 (Supplementary Fig. S3B). To confirm the link between HES1 and OGT, we evaluated gene expression of the phosphatase and tensin homolog (PTEN). PTEN expression is known to be negatively regulated by HES1^36^. In accordance with this, both the OGT knockdown and treatment with OSMI1 increased *PTEN* expression (Supplementary Fig. S3A). To our knowledge, this is the first instance where OGT has been shown to regulate PTEN.

In order to determine how inhibition of OGT causes down-regulation of HES1 in TNBC cells, we tested whether HES1 is modified with O-GlcNAc, using immuno-precipitation and confocal microscopy, but did not observe evidence of this (data not shown). Next, we assessed if inhibition of OGT affects transcriptional regulation of *HES1*, however, it did not affect *HES1* mRNA levels (Supplementary Fig. S3A). HES1 is an unstable protein, with a half-life of ~2 hours, undergoing continuous proteasomal degradation at the end of this period^37^. It has been reported that OGT inhibits proteasomal activity^38^, which prompted us to suggest that OSMI1 induces proteasomal degradation of HES1. Indeed, inhibition of the proteasome activity with MG-132 completely prevented down-regulation of HES1 in response to OSMI1 (Fig. 4C). This confirmed that OGT regulates HES1 protein stability through proteasomal degradation, enabling constitutive expression of this transcription factor.

Earlier we found that the combined cytotoxicity of OSMI1 and PR inhibitor was associated with a prominent increase in DNA damage (Fig. 2E,F). Inhibition of OGT enhanced the severity of DNA damage induced by MF in MCF7 cells. HES1 interacts with the fanconi anemia core complex, which facilitates the repair of DNA crosslinks^39^. We therefore hypothesized that inhibition of HES1 activity could promote DNA damage. HES1 expression is regulated by Notch signaling. Ligand-activated cleavage of Notch receptors by γ-secretase, and the following activation of down-stream signaling, can be blocked by γ-secretase inhibitor (GSI)^40^. Treatment with GSI alone or a knock-down of HES1 induced mild DNA damage, however, it was greatly potentiated by the simultaneous knock-down of OGT or treatment with OSMI1, respectively. In both cases the combined treatment resulted in a complete loss of HES1 (Supplementary Fig. S3E).

**Figure 5:**
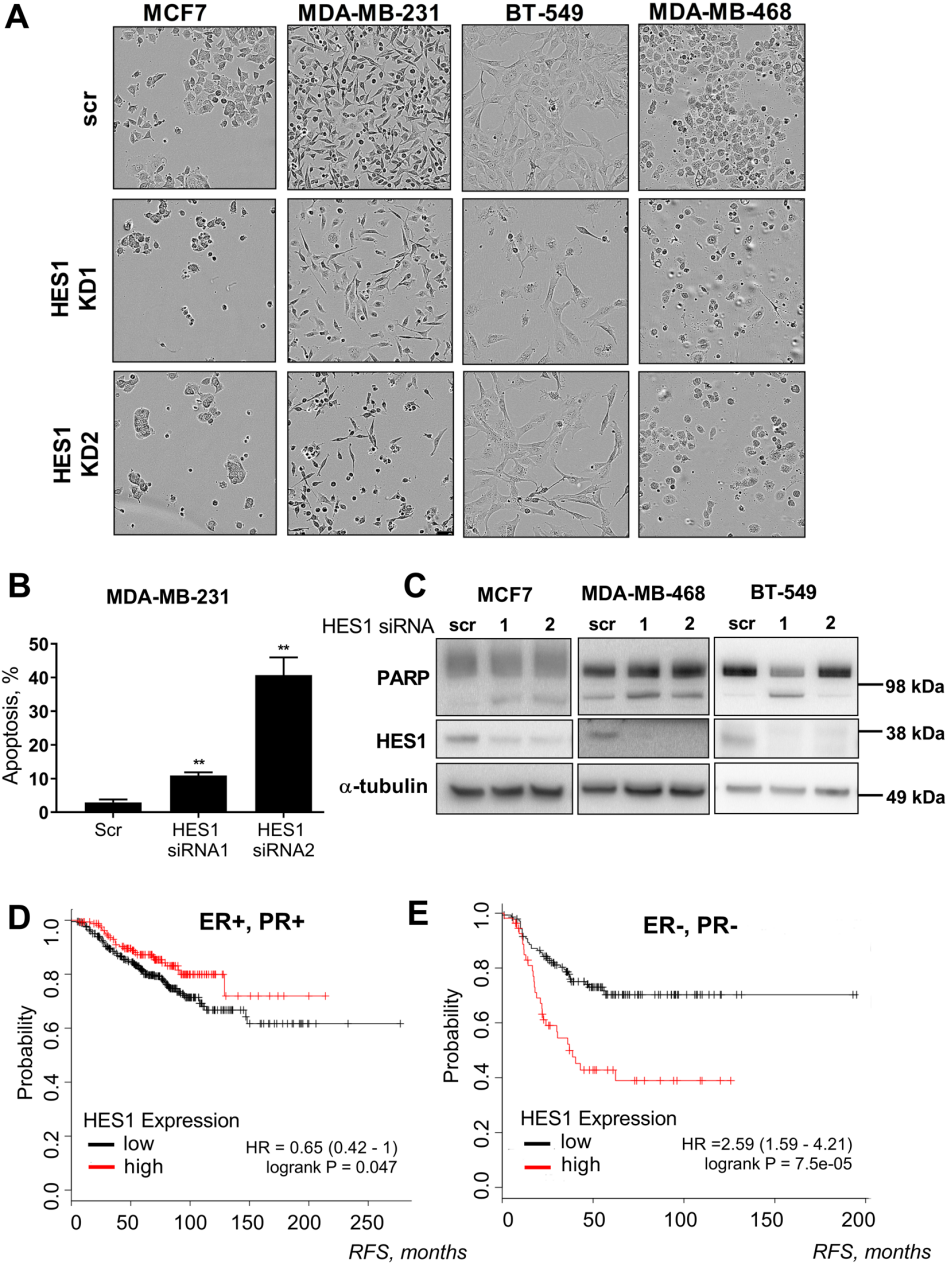
Loss of HES1 affects BC cell viability. (**A**) BC cells pictured after 72 hours of transient HES1 knock-down. (**B**) Apoptosis in MDA-MB-231 cells after 4 days of transient HES1 knock-down. Error bars - SEM, n = 3; **p < 0,005, unpaired t-test. (**C**) Total length and cleaved PARP and HES1 following 3 days of transient HES1 knock-down in MCF7, MDA-MB-468 and BT-549 cell lines. Representative of two biological replicates. (**D, E**) Relapse-free survival (RFS) in ER-positive, PR-positive (**D**), and ER-negative and PR-negative (**E**) breast cancer. Both cohorts excluded the HER-2-amplified BCs and patients that did not receive systemic treatment. Plots built using the online KM-plotter tool^24^.

Next, we moved on to assess whether targeting HES1 can replicate the cytotoxic effects observed after OGT inhibition. In the MDA-MB-231 cells, knock-down of HES1 potently induced apoptosis (Fig. 5B). To confirm that this response is not only specific to MDA-MB-231, we performed HES1 knock-downs in two additional TNBC cells lines, BT549 and MDA-MB-468, as well as in the MCF7 cells. Based on visual inspection, we observed fewer cells in response to the HES1 knock-down and possible induction of cell death (Fig. 5A). Indeed, the knock-down of HES1 induced PARP cleavage in all of the breast cancer cell lines tested (Fig. 5C). In addition, HES1 knockdown sensitized MCF7 cells to OSMI1 (Supplementary Fig. S3C,D). Taken together, these results suggest that HES1 is important for proliferation of both TNBC and receptor-positive cell lines.

In breast cancer, HES1 has been implicated in epithelial-to-mesenchymal transition^41^, stimulation of invasion^42^ and resistance to HER2 inhibitors^43^. In addition, HES1 has been shown to be important for proliferation of the TNBC cells^42^, which encouraged us to test if HES1 can be used as a prognostic marker. In patients with receptor-positive breast cancer, high level of HES1 expression correlates with longer relapse-free survival (Fig. 5D). In the receptor-negative breast cancers, however, high expression of HES1 was strongly associated with poor prognosis (Fig. 5E). Based on these data, we propose that HES1 has a distinct function in TNBC and in the hormone receptor-positive cells.

## Discussion

Abnormally high levels of O-GlcNAcylation in breast cancer have been linked to sustained proliferation^18^, invasion and metastasis^13^. However, these studies do not account for the characteristic diversity and heterogeneity of breast cancers. Here we found that OGT inhibition or knock-down is significantly more toxic to TNBC cells than to the hormone receptor-positive cells (Fig. 1C). While it is possible to sensitize receptor-positive cells to OGT inhibition by co-targeting either ER or PR, the combined effect of hormone receptor and OGT inhibition falls short of the prominent cyto-toxicity observed in the TNBC cells. This suggests that there are multiple contributing factors that dictate sensitivity of breast cancer cells to the decrease of protein O-GlcNAcylation.

Aiming to broaden our understanding of the response to OGT inhibition or knock-down, we performed a protein RPPA assay. Confirming previous reports that link OGT to the regulation of cell cycle (reviewed in^44^), we show that cell cycle phase distribution is affected upon OGT inhibition, leading to arrest in G1 phase (Fig. 3E,F). Notably, these changes were universal for the receptor-positive and -negative cell lines. In addition, we demonstrate that inhibition of OGT blocks the mTORC1 signaling. mTORC1 is a key regulator of protein synthesis, supporting up-regulated rate of translation in cancer tissues^45^. Earlier studies have detailed the cross-talk between OGT and mTORC1 in cancer cells, showing that de-regulation of either pathway has a similar effect on the other one^46–48^. Here we observed that OGT inhibition leads to a decrease in mTORC1 signaling in the TNBC cells, but not in the hormone receptor-positive MCF7 cells. This was apparent from a decrease in phosphorylation of PS6, indicating that p-PS6 level can serve as a biomarker of response to OGT inhibition in breast cancer cells.

OGT has been shown to limit the DNA damage response and facilitate DNA repair by directly inducing O-GlcNAcylation of H2AX and preventing its phosphorylation at the same site^14,15^. On its own, inhibition of OGT has led to modest DNA damage in all of the tested cell lines. This was greatly increased by additional treatment with MF in MCF7, and accompanied with prominent apoptosis (Fig. 2D–F). Here we found that OGT inhibition or knock-down leads to the proteasomal degradation of HES1 in the TNBC cells. Notch1-HES1 signaling is known to prevent DNA damage, while Notch1 inhibition or knock-down leads to accumulation of double-strand breaks after irradiation^49^. This response was shown to rely on HES1 interaction with the fanconi-anemia complex, which facilitates DNA repair^39^. In concert with these studies, simultaneous loss of OGT and HES1 resulted in extensive DNA damage in TNBC cells (Supplementary Fig. S3E). Our findings raise a possibility of targeting OGT to increase DNA damage in the TNBC cells.

In conclusion, we have established that OGT inhibition has a differential effect on breast cancer cells of different phenotypes, with TNBC cells having a particularly high sensitivity. We identify the transcriptional repressor HES1 and DNA damage as mediators of the response to OGT inhibition in these cells. The sheer complexity of OGT-mediated signaling makes it a challenging enzyme to study. More comprehensive breast cancer models are required to unravel the response to inhibition of OGT, and determine its usefulness for the treatment of TNBC.

## Supplementary information

Supplementary figures and supplementary figure legends.

## Data Availability

The patient survival datasets analyzed during the current study are available in the KM-plotter repository (http://kmplot.com/analysis/). The full RPPA results and the confocal microscopy images, showing the lack of O-GlcNAc modification on HES1, are available from the corresponding author on reasonable request. All other data generated or analyzed during this study are included in this published article and its Supplementary Files.
