# Supplementary material for: O-GlcNAc Transferase Inhibition Differentially Affects Breast Cancer Subtypes
Source: Sci Rep. 2019 Apr 5;9:5670. doi: 10.1038/s41598-019-42153-6 (PMC6450885; doi:10.1038/s41598-019-42153-6)
Supplement: Supplementary file 1 — Supplementary figures and supplementary figure legends. [file 41598_2019_42153_MOESM1_ESM.pdf]

O-GlcNAc Transferase Inhibition Differentially Affects Breast Cancer Subtypes

Anna Barkovskaya[1,2], Kotryna Seip[1], Bylgja Hilmarsdottir[1,3], Gunhild M. Maelandsmo[1,4], Siver A. Moestue[2,5] and Harri M. Itkonen[6*]

[1] Oslo University Hospital, The Norwegian Radium Hospital, Institute for Cancer Research, Department of Tumor Biology; Oslo, Norway
[2] NTNU - Norwegian University of Science and Technology, Department of Circulation and Medical Imaging; Trondheim, Norway
[3] Institute of Clinical Medicine, Faculty of Medicine, University of Oslo; Oslo, Norway.
[4] The Arctic University of Norway – University of Tromsø, Faculty of Health Sciences, Institute of Medical Biology; Tromsø. Norway
[5] Department of Health Sciences, Nord University; Bodø, Norway
[6] Department of Microbiology, Harvard Medical School, Harvard Institutes of Medicine; Boston, MA, USA

*Corresponding author

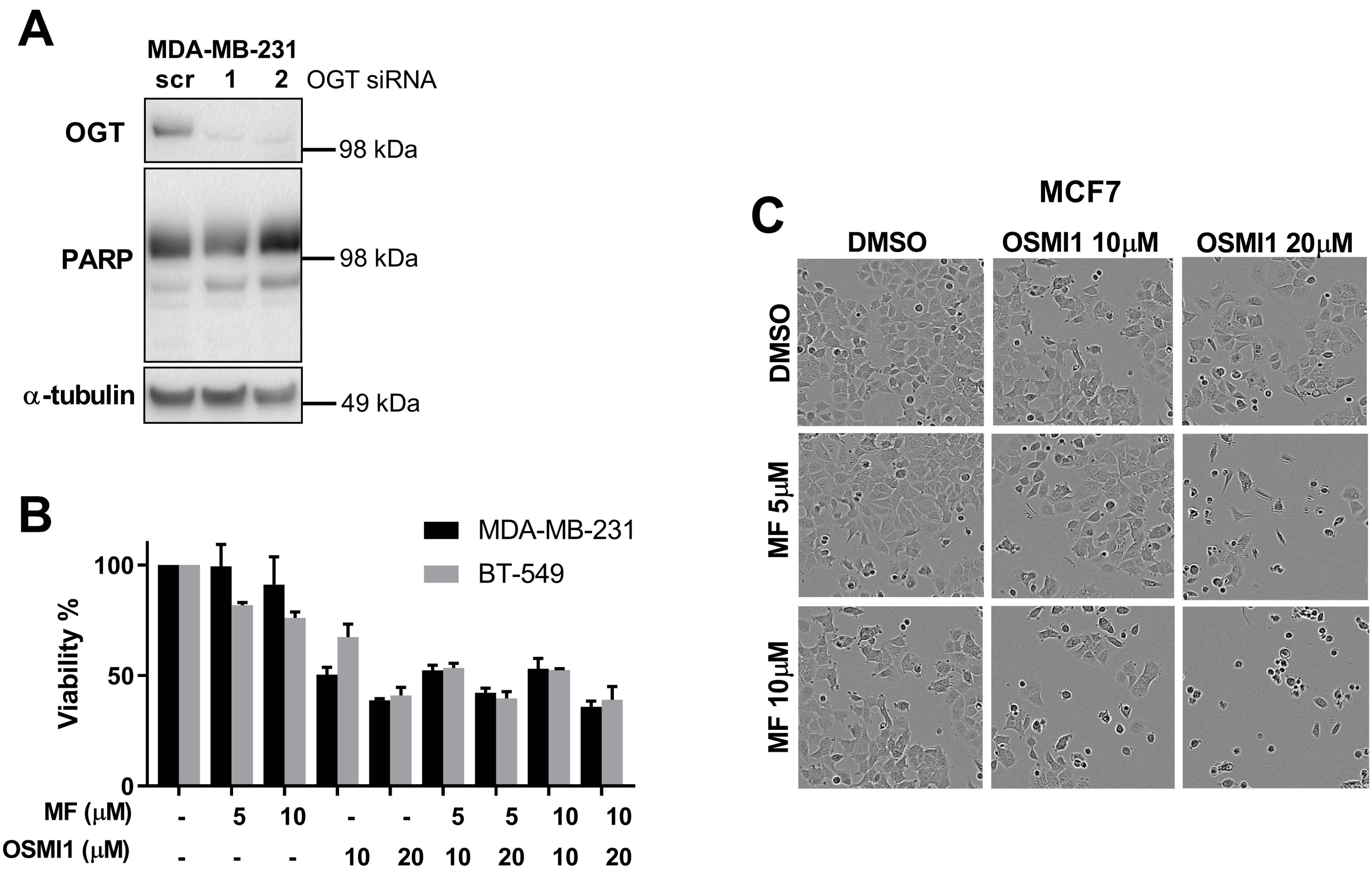

Figure 1. Supplementary.

A. Total length and cleaved PARP and OGT in MDA-MB-231 following 72 hours of OGT knockdown. **B.** Relative viability in TNBC cell lines following 72-hour long treatment with MF, OSMI1 or a combination. MTS assay. Error bars - SEM, n=3. **C.** Photographs of the MCF7 cells following 72 hours of treatment with MF, OSMI1 or a combination, taken with an Incucyte-FLR® instrument.

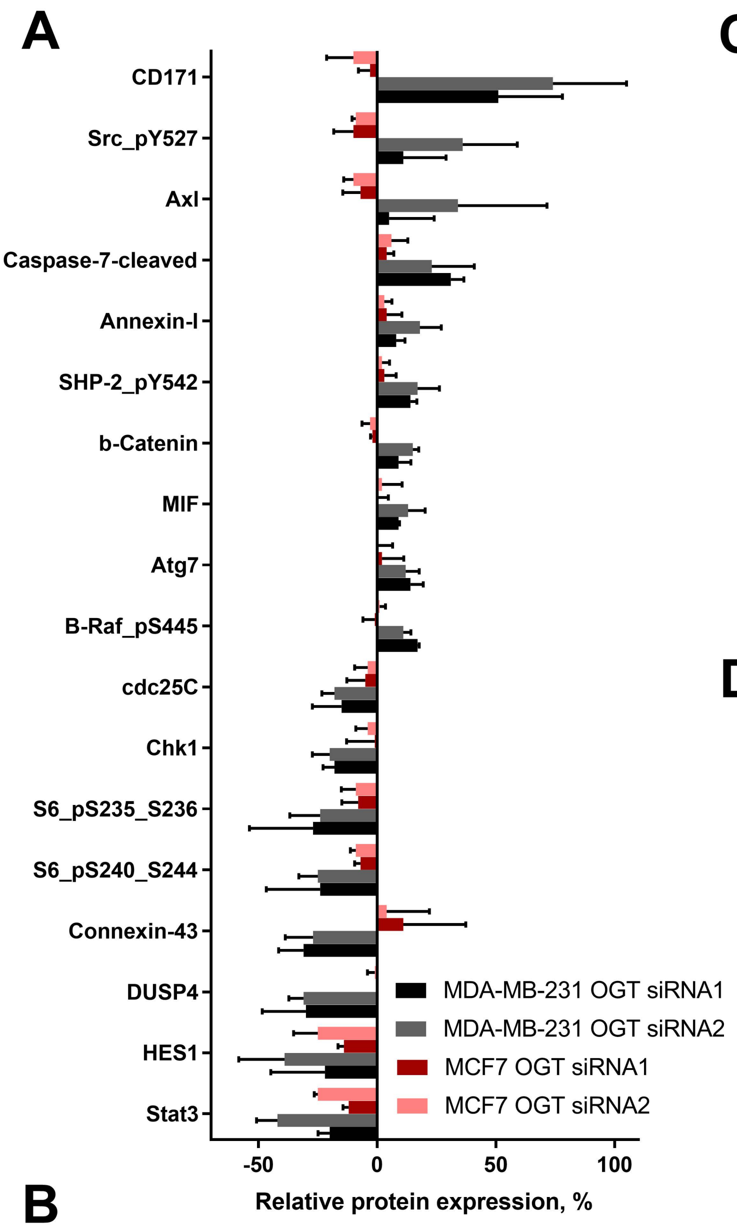

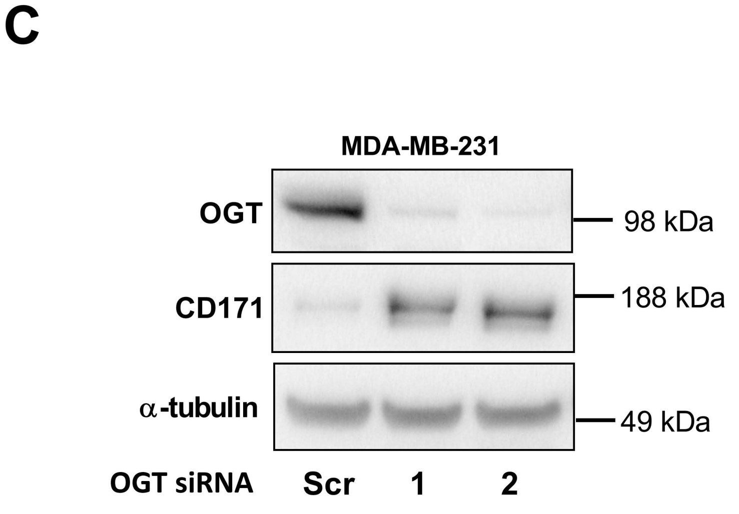

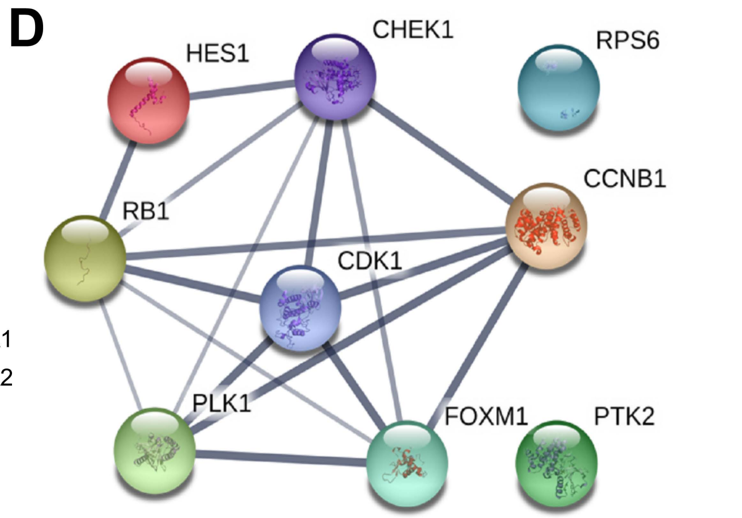

B

pathway description	*false discovery rate*
mitotic cell cycle phase transition	0.00018
regulation of protein metabolic process	0.00018
regulation of protein modification process	0.000227
G2/M transition of mitotic cell cycle	0.000308
G2 DNA damage checkpoint	0.000308
negative regulation of cell cycle	0.000308
regulation of cell cycle	0.000308
regulation of protein phosphorylation	0.000588
regulation of nucleobase-containing compound metabolic process	0.000588
regulation of cellular macromolecule biosynthetic process	0.000588

Figure 2. Supplementary.
A. Top up- and down-regulated proteins following 72 hours of transient siRNA OGT knock-down in MDA-MB-231 and the corresponding proteins in MCF7. RPPA array, error bars - SEM, n=3. **B.** List of top-regulated biological pathways following OSMI1 treatment in MDA-MB-231. STRING analysis. **C.** Validation of CD171 up-regulation following 72 hours of transient siRNA OGT knock-down in MDA-MB-231. **D.** STRING analysis of the top down-regulated proteins in MDA-MB-231 in the RPPA array after 24 hours of treatment with 20μM OSMI1. The width of connecting lines indicates strength of evidence supporting the connection between the proteins.

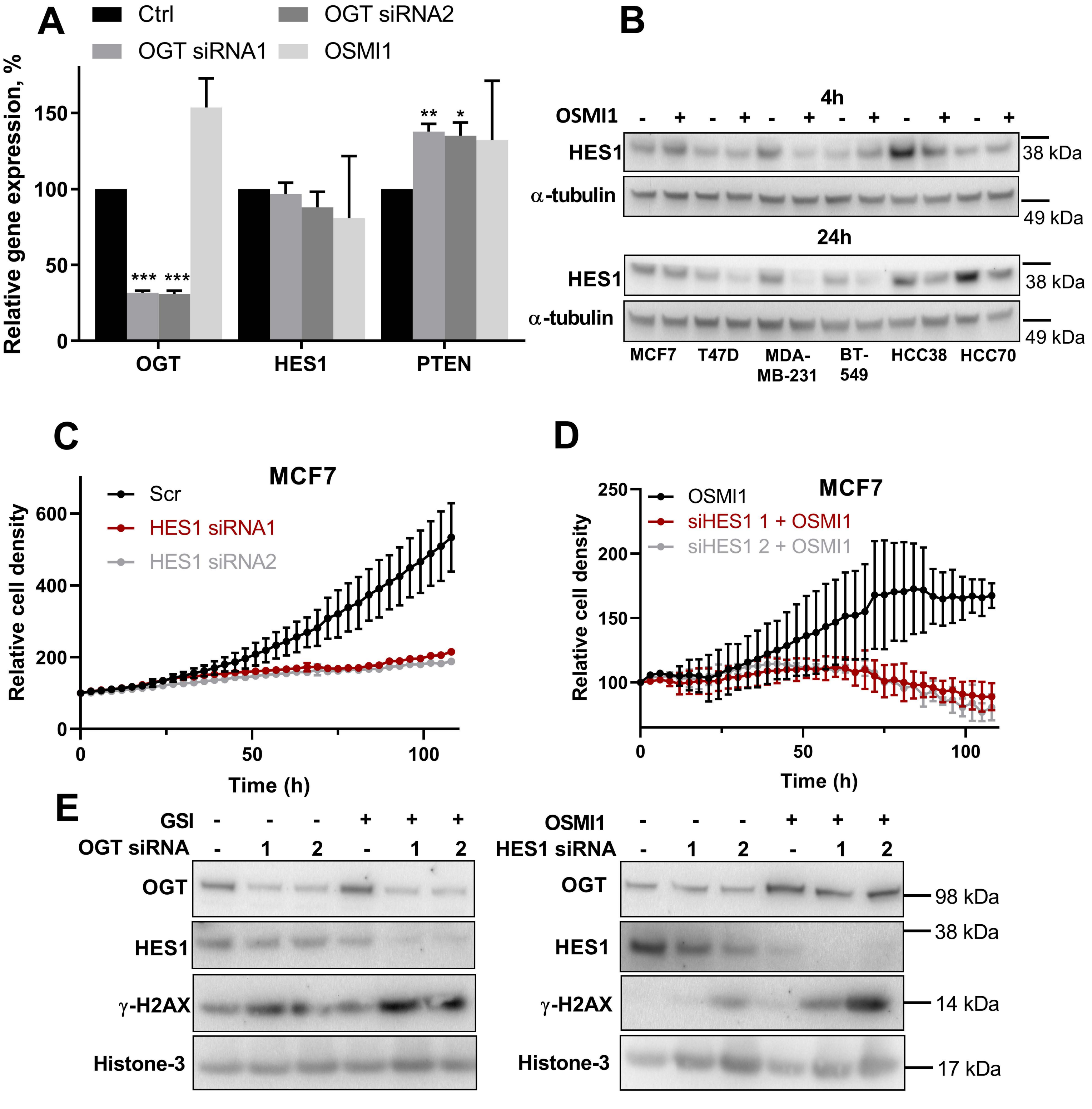

Figure 3. Supplementary.
A. Relative gene expression of OGT, HES1 and PTEN in MDA-MB-231 following 24 hours of treatment with 40μM OSMI1, or 72 hours of transient OGT knock-down. Error bars - SEM, n=3; *** - $p<0.001$, unpaired t-test. **B.** HES1 in the six BC cell lines following 4 and 24 hours of treatment with 40μM OSMI1. Representative of 2 separate experiments. **C, D.** Cell density, determined by the incucyte instrument, in MCF7 cells, treated with HES1 siRNA knock-down alone **(C)**, or in combination with 20μM OSMI1 **(D)**, where density measured at the first time point was assumed as 100%. Error bars - standard deviation, n=3. **E.** HES1, OGT and γ-H2AX in MDA-MB-231 following OGT or HES1 siRNA knock-down (90 hours), alone, or combined with either 10μM γ-Secretase inhibitor (GSI) or 20μM OSMI1 (72 hours). Representative of the three independent experiments.
